# Begomovirus-Associated Betasatellite Virulence Factor βC1 Attenuates Tobacco Defense to Whiteflies *via* Interacting With Plant SKP1

**DOI:** 10.3389/fpls.2020.574557

**Published:** 2020-08-27

**Authors:** Chi Zou, Yan-Ni Shu, Jing-Jing Yang, Li-Long Pan, Jing Zhao, Na Chen, Shu-Sheng Liu, Xiao-Wei Wang

**Affiliations:** Ministry of Agriculture, Key Laboratory of Molecular Biology of Crop Pathogens and Insects, Institute of Insect Sciences, Zhejiang University, Hangzhou, China

**Keywords:** tripartite interaction, tomato yellow leaf curl China virus/tomato yellow leaf curl China betasatellite, βC1, whitefly, plant defense response

## Abstract

Plant-mediated interactions between plant viruses and their vectors are important determinants of the population dynamics of both types of organisms in the field. The whitefly *Bemisia tabaci* can establish mutualism with begomoviruses *via* their shared host plants. This mutualism is achieved by the interaction between virulence factors and their host proteins. While the virulence factor βC1 encoded by tomato yellow leaf curl China betasatellite (TYLCCNB), a subviral agent associated to the begomovirus tomato yellow leaf curl China virus (TYLCCNV), may interact with plant protein MYC2, thereby establishing the indirect mutualism between TYLCCNV and whitefly, whether other mechanisms are involved remains unknown. Here, we found the *in vitro* and *in vivo* interactions between βC1 and tobacco protein S-phase kinase associated protein 1 (NtSKP1). Silencing the expression of *NtSKP1* enhanced the survival rate and fecundity of whiteflies on tobacco plants. NtSKP1 could activate the transcription of genes in jasmonic acid (JA) pathways by impairing the stabilization of JAZ1 protein. Moreover, βC1-NtSKP1 interaction could interfere JAZ1 degradation and attenuate the plant JA defense responses. These results revealed a novel mechanism underlying the better performance of whiteflies on TYLCCNV/TYLCCNB-infected plants.

## Introduction

Plants are constantly exposed to environmental biotic factors, including plant pathogens and herbivores, both of which are propelled into complex interaction networks in microecosystems (Rzanny and Voigt, 2012; Franco et al., 2017; Noman et al., 2020). Thereinto, virus-plant-vector tripartite interactions can impact the population dynamics of all the three members. In some cases, this tripartite interaction can become a determining factor in the outbreak of diseases and pests (Islam et al., 2020; Noman et al., 2020). For instance, cucumber mosaic virus, a non-persistently transmitted-virus, alters the performance of its vector aphid, like survival, fecundity, wing dimorphism, and behavior *via* host plants to optimize viral spread (Ziebell et al., 2011; Carmo-Sousa et al., 2014; Shi et al., 2016; Wu et al., 2017; Tungadi et al., 2020). Soybean mosaic virus infection of soybean plants may reduce plant palatability for its aphid vectors, but increase aphid feeding preference for infected plants, thus promoting its transmission (Peñaflor et al., 2016; Li et al., 2018). Although the biological significance of virus-plant-vector tripartite interactions has been appreciated, molecular mechanisms underlying these complex interactions remain largely unknown.

The genus *Begomovirus* (family *Geminiviridae*) is the most populous genus in plant viruses (Brown et al., 2015; Zerbini et al., 2017). Based on genomic organization, begomovirus may be divided into monopartite and bipartite begomoviruses. The genome of bipartite begomoviruses contain two single-stranded circular DNA molecules (DNA-A and DNA-B), while monopartite begomoviruses have only a single genomic component similar to DNA-A of bipartite begomoviruses. Some monopartite begomoviruses were found to be associated with satellite DNA, such as alphasatellites, betasatellites, or deltasatellites (Nawaz-Ul-Rehman and Fauquet, 2009; Zhou, 2013; Lozano et al., 2016; Zerbini et al., 2017). Begomoviruses have long time been a major constraint in agricultural production in tropical and subtropical regions around the globe (Mansoor et al., 2006; Perefarres et al., 2012; Rojas et al., 2018). Begomoviruses are exclusively transmitted by whiteflies (Hemiptera: Aleyrodidae) of the *Bemisia tabaci* complex, the abundance and distribution of which are major determinants of virus epidemics (Hogenhout et al., 2008; Navas-Castillo et al., 2011; Gilbertson et al., 2015; Whitfield et al., 2015; Islam et al., 2018). The *B. tabaci* complex contains at least 44 cryptic species, among which the invasive Middle East Asia Minor 1 (MEAM1) can efficiently transmit many begomoviruses (De Barro et al., 2011; Liu et al., 2012; Lee et al., 2013; Wei et al., 2014; Kanakala and Ghanim, 2019). In recent years, the relationships among begomoviruses, plants, and whiteflies has been explored extensively, which could be positive, neutral, and negative (Luan et al., 2014; Wang et al., 2017). For example, tomato yellow leaf curl China virus (TYLCCNV) infection of tobacco plants could increase the fecundity and longevity of *B. tabaci* MEAM1 (Jiu et al., 2007; Zhang et al., 2012; Luan et al., 2013; Li et al., 2014), while having no influence on *B. tabaci* MEAM1 on tomato, or negative influence on *B. tabaci* Asia II 3 on tomato (Liu et al., 2009).

TYLCCNV is a monopartite begomovirus, which is associated with tomato yellow leaf curl China betasatellite (TYLCCNB, genus *Betasatellite*, family *Tolecusatellitidae*) (Adams et al., 2017). TYLCCNB only encodes one protein βC1 (Cui et al., 2004). In recent studies, βC1 from betasatellites was found to enhance the symptoms of the helper viruses and suppress multiple host defense pathways, including transcriptional gene silencing, post-transcriptional gene silencing, and jasmonic acid (JA) signaling (Yang et al., 2011; Zhou, 2013). For example, TYLCCNB βC1 was found to subvert JA signaling pathway in plants through interacting with the transcription factor MYC2 protein, thereby increasing the performance of whiteflies (Zhang et al., 2012; Li et al., 2014). However, whether other mechanisms are involved in the manipulation of plant defense against whiteflies by TYLCCNB βC1 remains obscure.

JA, one of the most intensively studied plant hormones, has emerged as a major signaling molecule that integrates information perceived at the plant-insect interface into broad spectrum defense responses (Erb et al., 2012; Verma et al., 2016; Berens et al., 2017). JA can promote the accumulation of metabolites like indole-glucosinolates, camalexin, the non-protein amino acid Nδ-acetylornithine and terpenes that are detrimental to phloem-feeding insects, like green peach aphid (GPA) and whitefly (Mewis et al., 2005; Goggin, 2007; Louis and Shah, 2013; Zhao et al., 2019). Foliage spray of methyl jasmonate (MeJA) can improve plant resistance to whiteflies (Zhang et al., 2012). The JA-mediated production of terpenoids reduce performance of whiteflies on tobacco plants (Luan et al., 2013). The COI1-JAZ-MYC signaling complex, which is constituted by coronatine insensitive 1 (COI1), jasmonate ZIM-domain (JAZ), and helix-loop-helix transcription factor MYC2, is a central regulatory module in JA signaling cascade. Stress responsive jasmonoyl-L-isoleucine (JA-Ile) directly promotes the interaction between JAZ and COI1, leading to ubiquitination and degradation of JAZ protein *via* 26S proteasome, thereby derepressing MYC-mediated transcriptional reprogramming (Pauwels and Goossens, 2011; Zhang et al., 2017). Recently, Jia et al. (2016) found that cotton leaf curl Multan betasatellite (CLCuMuB) βC1 can interact with tobacco S-phase kinase associated protein 1 (SKP1) to subvert plant ubiquitination, thus promoting virus infection and disease symptom induction. However, whether TYLCCNB βC1 can interact with plant SKP1, increase the stability of plant JAZ proteins and suppress plant resistance to whiteflies remain unknown.

In this study, TYLCCNB βC1 was used as a bait to search for binding proteins in tobacco by yeast two-hybrid screening. We found that βC1 interacted with *Nicotiana tabacum* SKP1 protein (NtSKP1) and verified the interaction *via* bimolecular fluorescence complementation (BiFC) and GST pull-down. Then, bioassays were conducted to examine how βC1-NtSKP1 interaction impacts plant defense against whiteflies. Furthermore, transcript level of genes in JA signaling pathway and contents of JAZ1 protein were analyzed to explore the contribution of NtSKP1 to plant defense responses against whiteflies.

## Materials and Methods

### Plants, Virus, Betasatellite, and Whitefly

*Nicotiana tabacum* var. NC89, *N. benthamiana* line 16c, and *N. benthamiana* line H2B-RFP were provided by the Institute of Biotechnology, Zhejiang University. All plants used were grown in climate chamber at 26 ± 2°C under 14 h light/10 h dark cycle with 60% humidity. Infectious clones of TYLCCNV (Y10, GenBank Accession No.: AJ319675.1) and TYLCCNB (AJ781200) were described previously (Cui et al., 2004). A population of whitefly (Middle East Asia Minor 1, MEAM1) was collected in Hangzhou, China and maintained on cotton (*Gossypium hirsutum* L. cv. Zhemian 1793) in climate chamber. The purity of the culture was monitored every three generations using PCR-restriction fragment length polymorphism (PCR-RFLP) and *mtCO1* (GQ332577) sequencing analysis as described before (Qin et al., 2013).

### Plasmid Construction and Transformation

Coding sequences of *βC1* and *NtSKP1* were cloned into pClone007 for sequencing (TSINGKE, China). Plant expression constructs (p2YC-βC1, p2YN-NtSKP1, pCambia1305-NtSKP1-GFP, pCambia1305-βC1-GFP, pBIN2mDNA1-NtSKP1) and prokaryotic expression recombinant protein constructs (pGEX-6P-1-GST-βC1, pMAL-c5x-MBP-NtSKP1) were constructed using restriction endonuclease and T4 DNA ligase. Target fragments and plasmids were digested by specific restriction endonucleases (Thermo Scientific, USA) at 37°C for 2 h. Then, fragments were ligated to plasmids using T4 DNA ligase (Thermo Scientific, USA) at 22°C for 10 min.

Recombinant plasmids used for expression in plant were transformed into *Agrobacterium tumefaciens* (EHA105) by electroporation. Recombinant plasmids used for prokaryotic expression were transformed into *Escherichia coli* (BL21) using heat-shock method.

### Yeast Two Hybrid Assay

Forward primer (5′- CGCGGATCCATATGACTATCAAATACAA -3′) and reverse primer (5′-CCGCTCGAGTCATACATCTGAATTTG-3′) were used to amplify TYLCCNB *βC1*. Full-length of *βC1* was amplified by PCR, digested by BamHI and XhoI, then cloned into yeast vector pGBKT7. Plasmid pGBKT7-βC1 and tobacco cDNA library were co-transformed into *Saccharomyces cerevisiae* Gold strain according to Yeastmaker Yeast Transformation System 2 Kit (Clontech, USA). Transformants were allowed to grow on synthetic medium -Leu/-Trp at 30°C for 3 days and then transferred to selective medium -Leu/-Trp/-His/-Ade X-a-Gal/AbA. Five days later, positive clones were transferred into fresh YPDA liquids (30°C, 250 rpm) for 24 h. cDNA library plasmids in yeast positive clones were extracted and transformed into *E. coli* (DH5α) for sequencing.

Verification of the interaction between βC1 and NtSKP1 was performed according to Yeastmaker Yeast Transformation System 2 Kit (Clontech, USA). Forward primer (5′-CATATGATGTCCTCCTCAAAG-3′) and reverse primer (5′- GAATTCTCACTCAAATGCCCA-3′) were used to amplify *NtSKP1*. Full-length of *NtSKP1* was amplified by PCR, digested by NdeI and EcoRI, then cloned into pGADT7. Plasmid pGBKT7-βC1 and pGADT7-NtSKP1 were co-transformed into *S. cerevisiae* Gold strain. Transformants were grown on synthetic medium -Leu/-Trp at 30°C for 3 days and then transferred to selective medium -Leu/-Trp/-His/-Ade X-a-Gal/AbA.

### Agrobacterium-Mediated Transient Expression

Agrobacterium containing plant expression constructs (p2YC-βC1, p2YN-NtSKP1, pCambia1305- NtSKP1-GFP, pCambia1305-βC1-GFP, pBIN2mDNA1-NtSKP1) were grown in LB liquid medium (50 μg/ml kanamycin, 50 μg/ml rifampicin) at 28°C, 200 rpm until OD600 reached 0.6. Fresh agrobacterium cultures were centrifuged (4,000 rpm,10 min, room temperature) and resuspended in infiltration buffer (10 mM MgCl_2_, 10 mM MES, and 200 μM acetosyringone, pH 5.6) until OD600 reached 1.0. Then the agrobacterium was kept at room temperature for at least 3 h. Suspension liquid was infiltrated into whole leaf using a needleless syringe.

### Bimolecular Fluorescence Complementation (BiFC) Assay

Forward primer (5′-CCCTTAATTAACATGACTATCAAATACAACAA-3′) and reverse primer (5′-GGGACTAGTTACATCTGAATTTGTAAATA-3′) were used to amplify TYLCCNB *βC1*. Forward primer (5′-CCCTTAATTAACATGTCCTCCTCAAAGATGAT -3′) and reverse primer (5′-GGGACTAGTCTCAAATGCCCAAGCATTCT-3′) were used to amplify *NtSKP1*. Full-length of *βC1* and *NtSKP1* were digested by PacI and SpeI, then cloned into p2YC and p2YN vector respectively. Recombinant plasmids p2YC-βC1 and p2YN-NtSKP1 were transformed into agrobacterium respectively. Agrobacterium cultures containing p2YC-βC1 and p2YN-NtSKP1 were co-infiltrated into leaves of *N. benthamiana* line H2B-RFP. Next, 36-72 h after infiltration, RFP and YFP fluorescence were examined by Zeiss LSM710 confocal microscope.

### GST Pull-Down Assay

Forward primer (5′-CGCGGATCCATGACTATCA AATACAA-3′) and reverse primer (5′-CCGCTCGAGTCATACATCTGAATTTG-3′) were used to amplify TYLCCNB *βC1*. Forward primer (5′-ATAAGAATGCGGCCGCATGTCCTCCTCAA-3′) and reverse primer (5′-CGCGGATCCTCACTCAAATGCC-3′) were used to amplify *NtSKP1*. Full-length of *βC1* was digested by BamHI and XhoI, then cloned into vector pGEX-6P-1, and full-length of *NtSKP1* was digested by NotI and BamHI, then cloned into vector pMal-c5x. Then, GST-βC1 and MBP-NtSKP1 fusion proteins were expressed in *E. coli* (BL21). MBP-NtSKP1 was purified by Amylose resin (NEB), eluted with Elution buffer (20 mM Tris-HCl, 200 mM NaCl, 1 mM DTT, 1 mM EDTA, 10 mM maltose, PH=7.4) and desalted by disposable PD-10 desalting columns (GE Healthcare). GST-βC1 was purified by Glutathione Sepharose 4 Fast Flow (GE Healthcare) and then used to pull down MBP-NtSKP1 *in* *vitro* for 2 h at 4°C. The beads were washed five times with ice-cold 1×PBS. Then washed beads were boiled with SDS loading buffer for 10 min. Proteins were separated by SDS-PAGE and detected by western blot with anti-MBP tag mouse monoclonal antibodies (Abcam, UK).

### Phylogenetic Analysis

The amino acid sequences of SKP1 in different organisms were downloaded from NCBI database. These sequences were aligned by ClustalW. Phylogenetic analysis was conducted with MEGA 7.0 through neighbor-joining (NJ) method (1,000 replications).

### Subcellular Localization Assay

Forward primer (5′-CGCGGATCCATGTCCTCCTCAAAG-3′) and reverse primer (5′-CGAGCTCTCACTCAAATGCCCA-3′) were used to amplify *NtSKP1*. Full-length of *NtSKP1* was digested by BamHI and SacI, then cloned into the pCambia1305-GFP vector and transformed into agrobacterium. Agrobacterium clones expressing NtSKP1-GFP were infiltrated into leaves of *N. benthamiana* line H2B-RFP. Next, 36–72 h after infiltration, RFP and GFP fluorescence were observed by Zeiss LSM710 confocal microscope.

### Virus Induced Gene Silencing (VIGS) Assay

About 300 bp fragment of *NtSKP1* was amplified by forward primer (5′-CGCGGATCCCTACGATGTCCTCCTCAA-3′) and reverse primer (5′-TGCTCTAGATCAGAATCAAAGCCCTTA-3′). The fragment was digested by BamHI and XbaI, then cloned into the pBIN2mDNA1 vector, and the construct was transformed into agrobacterium. The method of VIGS was performed as previously described (Huang et al., 2011). After 15–20 days, total RNAs of tobacco plants were isolated to determine the silencing efficiency by qRT-PCR.

### qRT-PCR and Data Analysis

Total RNAs of tobacco plants were isolated using TRIzol^TM^ method. cDNA was synthesized using the PrimeScript^TM^ RT reagent Kit with gDNA Eraser (Takara, Dalian, China). qRT-PCRs were performed with the SYBR® Premix Ex Taq^TM^ II (Takara, Dalian, China) using BIO-RAD CFX96 PCR System (Bio-Rad, California, USA). *Glceraldehyde-3-phosphate dehydrogenase* (*GAPDH*) was used as internal control. The relative transcript levels of genes were calculated by the 2^-ΔΔCT^ method. Nucleotide sequences of the gene-specific primers were as follows: *NtSKP1* (forward primer, 5′-TGGCTGCCAACTACTTGAAC-3′; reverse primer, 5′-TCTCCTCTGGTGTCTTCCCT-3′); *MYC2b* (forward primer, 5′-ATCGGATGGGATGCTATGA-3′; reverse primer, 5′-GAAGCTGCTCTTGCGTGTA-3′); *PDF1.2* (forward primer, 5′- GGAAATGGCAAACTCCATGCG-3′; reverse primer, 5′-ATCCTTCGGTCAGACAAACG-3′); *GAPDH* (forward primer, 5′-GCAGTGAACGACCCATTTATCTC-3′; reverse primer, 5′-AACCTTCTTGGCACCACCCT-3′).

### Assessment of Plant Suitability to Whitefly

Newly emerged whiteflies (1–3 days) were used in bioassay. For the assessment of *NtSKP1*-silenced and control tobacco plants to whitefly, each cohort of five females and five males was released in a clip cage fixed on the abaxial surface of tobacco leaf. The number of live adults and total eggs laid were counted 7 days later. For the assessment of *NtSKP1*-overexpressed and control tobacco plants to whiteflies, number of live adults and total eggs whiteflies laid were counted 3 days after the release of whiteflies. Ten plants were used for each treatment in all experiments. The experiment was repeated three times.

### Plant Protein Extraction and Western Blot of JAZ1

Total proteins of each leaf sample were extracted as previously described (Ryu et al., 2007). About 0.1 g of leaf samples were triturated and then lysed with buffer (20 mM Tris-HCl, pH 7.0, 250 mM sucrose, 25% glycerol, 20 mM KCl, 2 mM EDTA, 2.5 mM MgCl_2_, 30 mM β-mercaptoethanol, protease inhibitor cocktail, and 0.7% Triton X-100). Leaf samples in buffer were centrifuged for 30 min at 4°C. The supernatant was used for western blot analysis.

Protein extracts were boiled with SDS loading buffer for 10 min, and proteins were separated by SDS-PAGE and detected with anti-JAZ1 antibodies as previously described (Li P. et al., 2019).

## Results

### βC1 Interacts With NtSKP1 *In Vivo* and *In Vitro*

**Figure 1:**
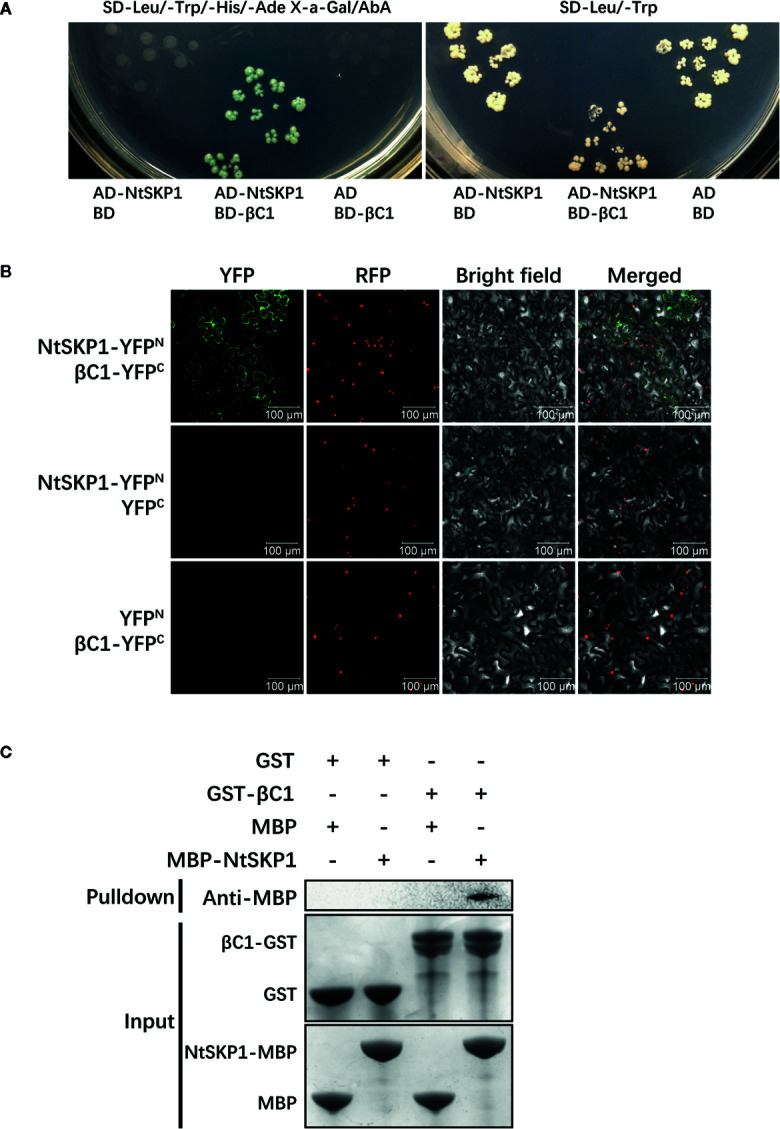
The interaction between βC1 and NtSKP1. **(A)** Interaction confirmation of βC1 and NtSBT1.7 *via* yeast two-hybrid system. Yeast strain Y2H Gold co-transformed indicated plasmids were grown on SD-Leu/-Trp/-His/-Ade X-a-Gal/AbA and SD-Leu/-Trp. Empty plasmids pGADT7/pGBKT7 were used as negative controls. **(B)** *In vivo* interaction confirmation of βC1 and NtSBT1.7 *via* BiFC analysis. βC1-YFP^C^ and NtSKP1-YFP^N^ were transiently co-expressed in *N. benthamiana* line H2B-RFP, of which nucleus were marked with RFP fusion protein. Photos were imaged at 48 h using a Zeiss LSM710 confocal microscope. Columns from left to right represent YFP fluorescence, RFP fluorescence, bright field, and YFP/RFP/bright field overlay. Empty plasmids were negative controls. Scale bars 100 μm. **(C)** *In vitro* interaction confirmation of βC1 and NtSBT1.7 *via* GST pull-down assay. Protein GST, βC1-GST, MBP, NtSKP1-MBP were expressed by prokaryotic expression, and purified by Glutathione agarose beads or Amylose resin. GST or GST-βC1 fusion proteins were used to pulldown MBP or MBP-NtSKP1 fusion proteins. Binding proteins were analyzed *via* SDS-PAGE and western blot assays using anti-MBP antibodies. At the start of Samples (Input) were stained by Coomassie blue solution. GST, MBP proteins were used as negative controls.

To further elucidate the mechanism underlying the plant-mediated indirect mutualism between TYLCCNV/TYLCCNB and whitefly, βC1 was used as a bait to screen the tobacco cDNA library by yeast two-hybrid system. NtSKP1, which is an indispensable subunit in plant Skp1-cullin-F-box (SCF) complex (**Figure 1A**), was captured. *NtSKP1* comprises a 468 bp open reading frame (ORF), encoding a 155 aa protein (≈17.53 kDa).

To verify the interaction between βC1 and NtSKP1, BiFC assay was performed *in vivo*. Agrobacterium containing βC1-YFP^C^ or NtSKP1-YFP^N^ were co-infiltrated into leaves of *N. benthamiana* (line H2B-RFP). Fluorescence confocal diagram showed strong YFP fluorescence in leaves expressing βC1-YFP^C^ and NtSKP1-YFP^N^ while no fluorescence was found in negative controls (**Figure 1B**), indicating the interaction between βC1 and NtSKP1 in plants.

Then, GST pull-down assay was performed to determine whether βC1 interacts with NtSKP1 *in vitro*. Fusion proteins GST-βC1 and MBP-NtSKP1 were expressed through prokaryotic expression. **Figure 1C** showed MBP-NtSKP1 could be captured by GST-βC1, and no signal was detected in negative controls.

### Phylogenetic Analysis, Domain Analysis, and Subcellular Localization of NtSKP1

**Figure 2:**
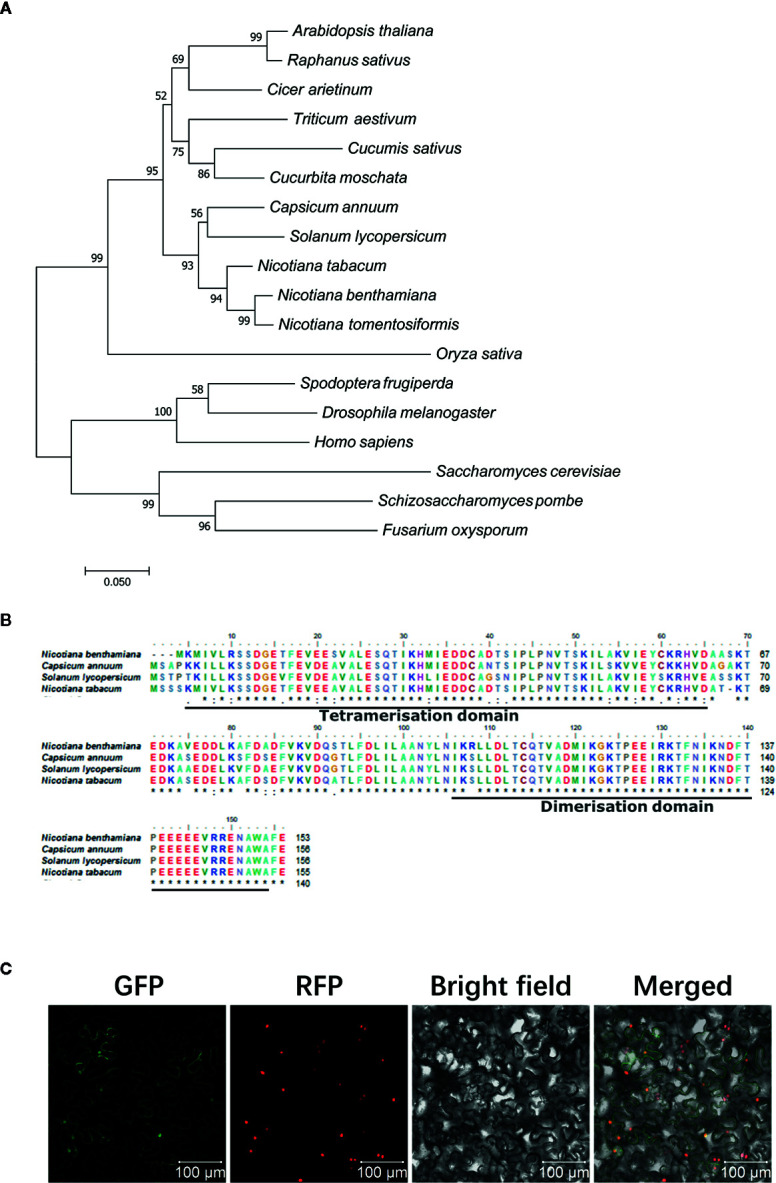
Phylogenetic analysis, sequence comparison, and subcellular localization of NtSKP1. **(A)** Phylogenetic tree analysis of SKP1. Accession numbers of the amino acid sequences included in the phylogenic tree as following as: *Nicotiana benthamiana* (AAO85510.1); common tobacco *Nicotiana tabacum* (XP_016435415.1); tobacco *Nicotiana tomentosiformis* (XP_009620406.1); radish *Raphanus sativus* (XP_018446355.1); chilly *Capsicum annuum* (AAX83944.1); tomato *Solanum lycopersicum* (ALA48968.1); *Arabidopsis thaliana* (NP_565123.1); wheat *Triticum aestivum* (AIJ50267.1); cucumber *Cucumis sativus* (XP_004146156.1); squash *Cucurbita moschata* (XP_022923340.1); Cicer arietinum (NP_001352203.1); rice *Oryza sativa* (AAT09201.1); fall armyworm *Spodoptera frugiperda* (AGP04177.1); fruit fly *Drosophila melanogaster* (NP_001284 755.1); human *Homo sapiens* (NP_008861.2); brewer’s yeast *Saccharomyces cerevisiae* (NP_010615.3); fission yeast *Schizosaccharomyces pombe* (BAB62325.1); *Fusarium oxysporum* (AAT85970.1). These sequences were aligned by ClustalW. Phylogenetic analysis was conducted with MEGA 7.0 through neighbor-joining (NJ) method (1,000 replications). **(B)** Amino acid sequence alignment and domain analysis of SKP1 proteins among *Nicotiana benthamiana*, *Capsicum annuum*, *Solanum lycopersicum*, and *Nicotiana tabacum*. **(C)** Subcellular localization of NtSKP1. Fusion protein NtSKP1-GFP were transiently expressed in *N. benthamiana* line H2B-RFP, of which nucleus were marked with RFP fusion protein. Photos were imaged at 48 h using a Zeiss LSM710 confocal microscope. Columns from left to right represent GFP fluorescence, RFP fluorescence, bright field, and GFP/RFP/bright field overlay. Scale bars 100 μm.

SKP1 commonly exists in eukaryotic organisms (Kong et al., 2004). Phylogenetic tree analysis showed that phylogenetically NtSKP1 was relatively closer to that in *N. benthamiana* and *N. tomentosiformis* than other plants, and it differed greatly from other organisms, such as microorganism or insects (**Figure 2A**). As a bridge between Cullin and F-box protein, SKP1 was composed of two typical conserved domains: tetramerisation domain and dimerisation domain (**Figure 2B**). The subcellular localization assay showed that protein NtSKP1 accumulated in nucleus and cytoplasm (**Figure 2C**).

### NtSKP1 Contributes to Plant Immunity Against Whitefly

**Figure 3:**
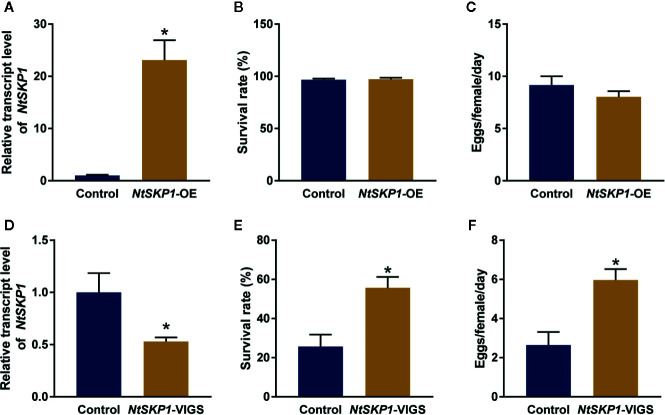
NtSKP1 regulates tobacco defense against whitefly. **(A)** Relative transcript levels of *NtSKP1* in control and *NtSKP1*-overexpressed (*NtSKP1*-OE) tobacco plants. GFP or NtSKP1-GFP fusion proteins were transiently expressed in *N. tabacum* var. NC89. Total RNAs were extracted from each plant respectively at 48 h and subjected to qRT-PCR to quantify the mRNA levels of *NtSKP1*. *GAPDH* was used as the internal reference. Values are mean ± SEM, n = 5. **(B)** Survival rates of whiteflies on control and *NtSKP1*-overexpressed tobacco plants. GFP or NtSKP1-GFP fusion proteins were transiently expressed in *N. tabacum* var. NC89. Each cohort of five females and five males was released in a clip cage fixed on the abaxial surface of tobacco leaf at 48 h. The numbers of live adults were counted 3 days later. Values are mean ± SEM, n = 20. **(C)** Average numbers of eggs one day laid by per female whitefly on control and *NtSKP1*-overexpressed tobacco plants. GFP or NtSKP1-GFP fusion proteins were transiently expressed in *N. tabacum* var. NC89. Each cohort of five females and five males was released in a clip cage fixed on the abaxial surface of tobacco leaf at 48 h. The numbers of eggs were counted 3 days later. Values are mean ± SEM, n = 20. **(D)** Relative transcript levels of *NtSKP1* in control and *NtSKP1*-silenced (*NtSKP1*-VIGS) tobacco plants. Gene silencing was achieved by VIGS assay. Total RNAs were extracted from each plant respectively and subjected to qRT-PCR to quantify the mRNA levels of *NtSKP1*. *GAPDH* was used as the internal reference. Values are mean ± SEM, n = 5. **(E)** Survival rates of whiteflies on control and *NtSKP1*-silenced tobacco plants. Gene silencing was achieved by VIGS assay. Each cohort of five females and five males was released in a clip cage fixed on the abaxial surface of tobacco leaf. The numbers of live adults were counted 7 days later. Values are mean ± SEM, n = 30. **(F)** Average numbers of eggs one day laid by per female whitefly on control and *NtSKP1*-silenced tobacco plants. Gene silencing was achieved by VIGS assay. Each cohort of five females and five males was released in a clip cage fixed on the abaxial surface of tobacco leaf. The numbers of eggs were counted 7 days later. Values are mean ± SEM, n = 30. Student’s t test was used for significant difference analysis (*P < 0.05).

To investigate the function of NtSKP1 in plant defense against whitefly, *NtSKP1* was transiently expressed in tobacco plants (**Figure 3A**). The survival rate and fecundity of whiteflies had no significant differences between *NtSKP1*-overexpressed tobacco plants and control plants (**Figures 3B, C**). These results revealed that the overexpression of *NtSKP1* did not affect plant defense response against whiteflies. However, when *NtSKP1* was knocked down *via* VIGS (**Figure 3D**), the survival rate and fecundity of whiteflies on *NtSKP1*-silenced tobacco plants were significantly higher than control plants (**Figures 3E, F**). These results indicated that NtSKP1 positively regulated plant defenses against whiteflies.

### NtSKP1 Modulates the Transcript Level of Genes in JA Signaling Pathway

**Figure 4:**
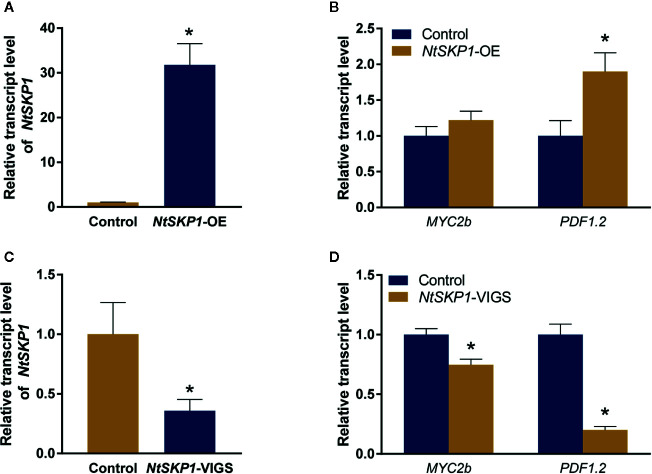
NtSKP1 affects the transcription of genes in JA signaling pathway. **(A)** Relative transcript levels of *NtSKP1* in control and *NtSKP1*-overexpressed (*NtSKP1*-OE) tobacco plants. Values are mean ± SEM, n = 6. **(B)** Relative transcript levels of *MYC2b* and *PDF1.2* in control tobaccos (Control) and *NtSKP1*-overexpressed (*NtSKP1*-OE) tobaccos. Values are mean ± SEM, n = 6. **(C)** Relative transcript levels of *NtSKP1* in control and *NtSKP1*-silenced (*NtSKP1*-VIGS) tobacco plants. Values are mean ± SEM, n = 6. **(D)** Relative transcript levels of *MYC2b* and *PDF1.2* in control tobaccos (Control) and *NtSKP1*-silenced (*NtSKP1*-VIGS) tobaccos. Values are mean ± SEM, n = 6. Total RNAs were extracted from each plant respectively and subjected to qRT-PCR to quantify the mRNA levels of target genes. *GAPDH* was used as the internal reference. Student’s t test was used for significant difference analysis (*P < 0.05).

SCF complex functions as a central regulator in JA signaling (Marino et al., 2012; Shu and Yang, 2017; Kelley, 2018). As JA functions in plant defense against whiteflies (Sun et al., 2017; Zhang et al., 2018), we wondered whether NtSKP1 impacted the JA signaling pathway in tobacco. Thus, transcript level of JA signaling pathway gene *MYC2b* and plant defensin gene *PDF1.2* was measured by RT-qPCR. Our results showed that the transcript level of *PDF1.2* was induced in *NtSKP1*-overexpressed plants (**Figures 4A, B**). In *NtSKP1*-silenced plants, the transcript level of *MYC2b* and *PDF1.2* was down-regulated (**Figures 4C, D**). Taken together, these results showed that NtSKP1 participated in regulating JA signaling pathways, thereby promoting whitefly performance.

### βC1 Targets NtSKP1 to Regulate Plant Immunity Through Interfering With JAZ1 Degradation

**Figure 5:**
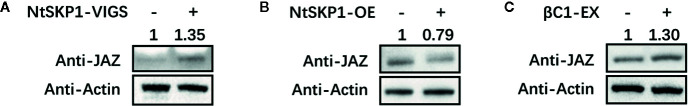
NtSKP1 and βC1 interfere the degradation of JAZ1 protein. **(A)** Western blot assay of JAZ1 protein in *NtSKP1*-silenced (NtSKP1-VIGS) tobacco plants. Gene silencing was achieved by VIGS assay. **(B)** Western blot assay of JAZ1 protein in *NtSKP1*-overexpressed (NtSKP1-OE) tobacco plants. GFP or NtSKP1-GFP fusion proteins were transiently expressed in *N. tabacum* var. NC89 for 48 h. **(C)** Western blot assay of JAZ1 protein in *βC1*-expressed (βC1-EX) tobacco plants. YFP^C^ or βC1-YFP^C^ fusion proteins were transiently expressed in *N. tabacum* var. NC89 for 48 h. Total plant protein extracts were analyzed *via* SDS-PAGE and western blot assays using anti-JAZ antibodies. Actin was the internal reference. Intensity was detected by image J.

JA-mediated responses are restrained by JAZ proteins, which are transcription repressors through binding to and repressing the activity of MYCs (Zhang et al., 2017). NtSKP1 is a component of SCF complex and plays an important role in ubiquitination-mediated protein degradation (Zeng et al., 2006; Shu and Yang, 2017; Zhou and Zeng, 2017; Serrano et al., 2018). We examined whether NtSKP1 took part in the turnover of JAZ1, thus modulating JA signaling pathway. **Figure 5A** showed that JAZ1 protein had higher accumulation in *NtSKP1*-silenced plant. In *NtSKP1*-overexpressed plant, the accumulation of JAZ1 was lower than control plant (**Figure 5B**). Similarly, JAZ1 was more stable in *βC1*-expressed plant (**Figure 5C**). Taken together, βC1 might interfere the degradation of JAZ1 through interacting with NtSKP1, thus suppressing the plant JA signaling pathway.

## Discussion

**Figure 6:**
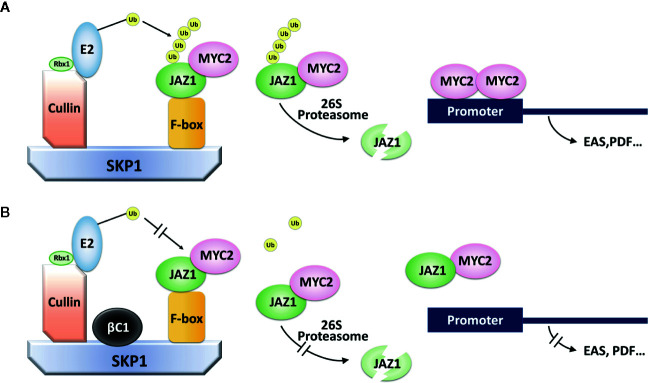
A model for the role of βC1 in regulating plant defense reaction. **(A)** The signaling pathway of SCF complex in plants. SCF complex is one kind of E3 ligase. E2-Ub complex can interact with E3 ligase *via* the help from Rbx1, making the E3 protein substrates (JAZ1) carry with polyubiquitin chain. Then, JAZ1-Ub chain is recognized by 26S proteasome, eventually degraded, releasing MYC2 transcription factor to activate genes expression in plant defense. **(B)** The pathway of SCF complex when plants were infected by TYLCCNV. βC1 can bind to SKP1 protein and interfere the normal function of SCF complex. JAZ1-MYC2 was stable and genes expression in plant defense were not activated.

Betasatellites associated to begomoviruses have been shown to be determinants in virus disease symptom induction, virus movement, repression of plant defense, and other aspects of viral pathogenesis (Zhou, 2013). βC1 interacts with a number of host proteins to affect phytohormone, methylation, ubiquitin, and photosynthesis pathways in plants (Gnanasekaran et al., 2019; Yang et al., 2019). Here, we found that TYLCCNB βC1 interacted with NtSKP1 to disrupt the integrality of SCF complex, thereby suppressing JAZ1 degradation and the subsequent activation of JA signaling pathways (**Figure 6**).

SKP1 has been shown to be a target for several plant pathogen effectors, such as P7-2 from rice black streaked dwarf virus (Tao et al., 2017), βC1 from CLCuMuB (Jia et al., 2016), and P0 from brassica yellows virus (Li Y. et al., 2019). These pathogen effectors modulate plant immunity *via* damaging the integrity of SCF complex, thus establishing a successful infection. However, the role of SKP1 or SCF complex in plant defense against insects have not been proved yet. Kottapalli et al. (2006) found *SKP1* was in eight tandem repeats on chromosome 9 and responsible for gall midge resistance phenotype. Hence, *SKP1* may be a candidate gene in rice for gall midge insect resistance. In this study, we found the *in vivo* and *in vitro* interaction between TYLCCNB βC1 and NtSKP1 (**Figure 1**), and we confirmed that whiteflies performed better on *NtSKP1*-silenced tobacco plants (**Figures 3E, F**). Higher survival rate of whitefly adults and more offsprings on plant can in turn facilitate the spread of begomoviruses. However, the overexpression of NtSKP1 did not affect the survival rate and fecundity of whiteflies (**Figures 3B, C**). Perhaps the background expression of NtSKP1 is sufficient to achieve its function in plants, or JA signaling pathway have some mechanisms to alleviate or balance the overexpression of NtSKP1. These results showed a novel function of NtSKP1 in plant defense against insect herbivores, and the involvement of NtSKP1 in TYLCCNV/TYLCCNB-whitefly mutualism.

Previous studies have been conducted to investigate the role of JA in TYLCCNV/TYLCCNB-plant-whitefly tripartite interactions. TYLCCNV can inhibit the JA-biosynthesis genes *FAD3* and *FAD7*, as well as JA-regulated gene *PDF1.2*. Also, it reduces the JA level through a SA-independent mechanism in tobacco plants (Zhang et al., 2012). TYLCCNB βC1 can interact with MYC2, a transcription factor in JA signaling pathway, to suppress the synthesis of tobacco terpenoids, thus improving the performance of whiteflies (Luan et al., 2013; Li et al., 2014). In this study, we found NtSKP1 contributed to JA signaling pathway and regulated the transcription of *MYC2b* and *PDF1.2* (Fig 4). Furthermore, SCF complex, which consisted by SKP1, Cullin, and F-box proteins, is a kind of ubiquitination E3 ligase, and it plays prominent roles in the perception of many phytohormones including JA, gibberellin, ethylene, salicylic acid, and strigolactones (Yu et al., 2007; Kelley, 2018). SCF complex in *Arabidopsis* is essential for JA signaling, and it leads to the ubiquitination and degradation of JAZ protein *via* 26S proteasome to derepress MYC-mediated transcriptional reprogramming (Xu et al., 2002; Zhang et al., 2017). In this study, we verified NtSKP1 could impact the stabilization of JAZ1 protein (**Figure 5**), which might explain the role of SKP1 in JA signaling pathway. These results demonstrated that TYLCCNB βC1 might implement more than one strategy to suppress plant defense responses, and finally achieve the mutualism between TYLCCNV/TYLCCNB and whiteflies.

Taken together, our study uncovers a novel mechanism underlying the manipulation of whitefly-plant interactions by a begomovirus-associated betasatellite. The findings in this study not only add to our knowledge of βC1 function in modulating plant physiology, but also help to elucidate the nature of whitefly-plant interactions.

## Data Availability Statement

All datasets presented in this study are included in the article/supplementary material.

## Author Contributions

CZ designed and carried out the experiments, analyzed data, and wrote the first draft of the manuscript. Y-NS did the protein interaction verification assay and did the bioassays of the whitefly. J-JY and NC did the bioassays of the whitefly. L-LP, JZ, S-SL, and X-WW conceived the study and revised the manuscript.

## Funding

Financial support for this study was provided by the National Natural Science Foundation of China (31930092, 31925033) and the Zhejiang Provincial Natural Science Foundation of China (LZ20C140003).

## Conflict of Interest

The authors declare that the research was conducted in the absence of any commercial or financial relationships that could be construed as a potential conflict of interest.
